# Influence of Carbon Nanotube Coatings on Carbon Fiber by Ultrasonically Assisted Electrophoretic Deposition on Its Composite Interfacial Property

**DOI:** 10.3390/polym8080302

**Published:** 2016-08-17

**Authors:** Jianjun Jiang, Chumeng Xu, Yang Su, Qiang Guo, Fa Liu, Chao Deng, Xuming Yao, Linchao Zhou

**Affiliations:** Shaanxi Engineering Research Center for Digital Manufacturing Technology, Northwestern Polytechnical University, Xi’an 710072, China; tancy601@163.com (C.X.); syang2022@163.com (Y.S.); guoqiang2014@mail.nwpu.edu.cn (Q.G.); liufa2013@mail.nwpu.edu.cn (F.L.); 18509264418@163.com (C.D.); yaoxuming@gmail.com (X.Y.); zhoulinc2014@163.com (L.Z.)

**Keywords:** ultrasonic, carbon nanotubes, carbon fibers, electrophoretic deposition, mechanical properties

## Abstract

Carbon nanotube (CNT) coatings were utilized to enhance the interfacial properties of carbon fiber (CF)/epoxy(EP) composites by ultrasonically assisted electrophoretic deposition (EPD). A characterization of the CF surface properties was done before and after coating (surface chemistry, surface morphologies, and surface energy). The result shows that oxygenated groups concentrations of the CF surfaces experienced significant increases from 12.11% to 24.78%. Moreover, the uniform and homogeneous CNT films were tightly attached on the surface of CF, and the surface wettability of CF is significant improved by enhanced surface free energy when introduced ultrasonic during the EPD process. In addition, the interlaminar shear strength (ILSS) and water absorption of CF/EP composite were measured. Scanning electron microscopy (SEM) revealed that the fracture mechanisms of the new interface layer formed by depositing CNTs on the CF surface contributed to the enhancement of the mechanical performance of the epoxy. This means that the efficient method to improve interfacial performance of composites has shown great commercial application potential.

## 1. Introduction

Carbon fiber/epoxy (CF/EP) composites materials are widely used in automobile, aviation and aerospace fields due to their tailorability, high strength, and light weight [1,2]. The interface strength of CF and EP determines the mechanical properties of CF/EP composites. However, employing CF as reinforcements in advanced composites has been limited because of the surface of untreated carbon fiber that has large surface inertness and lack of reactive functional groups [3,4,5,6]. Therefore, it is necessary to improve the fiber-matrix adhesion to enhance mechanical properties.

Carbon nanotubes (CNTs) have drawn enough attention on enhancing composites due to their favorable mechanical properties [7,8,9]. Grafting CNTs onto fiber surface is a practical method to improve fiber surface area, forming mechanical interlocking on the interface between CF and EP, which may improve stress transfer and interfacial properties [10]. So far, a lot of studies have been devoted to introducing CNTs on the surface of CF to improve the interfacial properties of composites, such as chemical vapor deposition (CVD) [11] and electrophoretic deposition (EPD) [12,13]. Both methods could successfully introduce CNTs on the surface of CF with strong adhesion. However, CVD uses high temperatures and predeposited catalysts, seriously limiting the practical application. EPD is known to be one of the efficient techniques to produce large-scale reinforcement of nano-particles in composite applications, which exhibit obvious advantages over other surface coating process, such as low process and low-complexity of the deposited thickness [14,15,16].

Our previous work found that the stability and homogeneity of the CNT deposited films cannot be obtained caused by water electrolysis, for solving this problem, ultrasonication was applied during the electrophoretic deposition process [17,18]. In this study, special emphasis has been put on the influence of CNT coatings on carbon fiber by ultrasonically assisted electrophoretic deposition on its composite interfacial property. Therefore, surface morphologies and surface roughness of the fibers were examined using scanning electron microscopy (SEM) and atomic force microscopy (AFM), changes in surface chemistry were studied using X-ray photoelectron spectroscopy (XPS). Surface free energy and contact angle of the fibers were characterized by dynamic contact angle analysis (DCAA), the humidity resistance of composites were tested by the water absorption, and the mechanical properties of composites were evaluated by interlaminar shear strength (ILSS). Fracture mechanisms of the composite were examined by scanning electron microscopy.

## 2. Materials and Methods

### 2.1. Materials and Processing

The unidirectional CF layers were comprised of Polyacrylonitrile (PAN)-based 12K carbon fibers that have areal density of 300 g/cm^2^, as provided by the vendor. (Toray, Tokyo, Japan). Carbon nanotubes (95% purity, diameter about 10–20 nm, length 10–30 μm) were obtained from Nanjing Xianfeng. Port. Co., Ltd. (Nanjing, China). The epoxy resin was bought from Yueyang Chemical Reagent Co., Ltd. (Yueyang, China). The curing agent was supplied by Shanghai Jingchun Chemical Reagent Co., Ltd. (Shanghai, China).

The CF layers were refluxed in acetone at 80 °C for 72 h, then washed with deionized water repeatedly and dried under vacuum at 80 °C for 3 h to remove sizing agent (labeling as desized CF).

The carbon nanotubes were refluxed in a 3:1 (*v*:*v*) mixture of concentrated sulfuric acid and nitric at 100 °C for 4 h to introduce carboxylic acid groups, then diluted with deionized water, suction filtering, and deionized water washing to neutral. The acid-treated CNTs were dispersed for 3 h in deionized water by ultrasonic to obtain dispersions of 0.5 g/L.

### 2.2. Preparation of Carbon Nanotubes (CNTs)/Carbon Fiber (CF) Hybrid Fiber Composites

**Figure 1:**
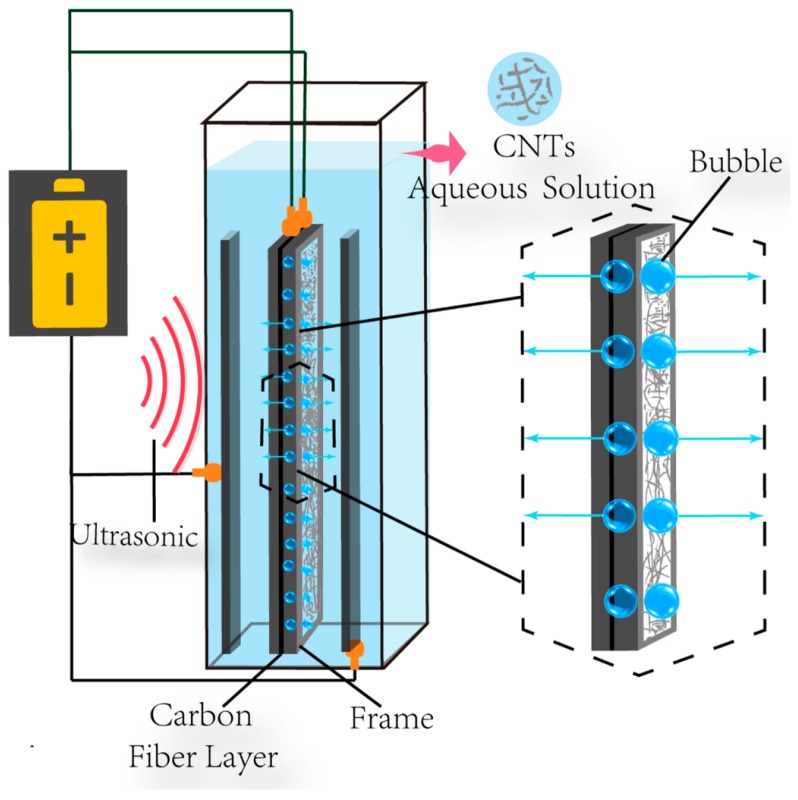
Schematic for ultrasonically assisted electrophoretic deposition (EPD) process of carbon nanotubes (CNTs) onto carbon fiber.

A schematic of the ultrasonically assisted EPD setup is shown in Figure 1. For the EPD of CNTs onto the CF layers, carbon fiber layer of 50 × 20 cm^2^ with opposing stainless steel plates electrodes placed was used as the deposition electrode, and two graphite plates were used as the counter electrodes. pH = 10 was used as an EPD condition of the CNT aqueous dispersion and CNT concentration was 0.25 g/L. The pH 10.0 aqueous solution was obtained by adding a 0.1 M sodium hydroxide solution. In order to obtain 0.25 g/L CNT dispersions, we disperse CNTs in deionized water by ultrasonication. The EPD process was carried out under a constant voltage of 20 V for 20 min; Ultrasonic assistance was provided by sonicator (60 W, 40 KHz). For ultrasonically assisted EPD, ultrasound was applied constantly during EPD.

Carbon fiber fabrics were used as the base material to synthesize the CNTs/CF/EP hybrid composites. The CNTs/CF preforms were infiltrated with epoxy using vacuum-assisted resin transfer molding (VARTM) with the epoxy and curing agent at a ratio of 100/33 parts by weight. In the VARTM process, the epoxy was infused into the carbon fibers under vacuum. After the infiltration of epoxy, the composites were cured for 12 h at 80 °C.

### 2.3. Characterizations

The morphology and thickness of the CNTs were observed on TEM (Tecnai-F30-G^2^, FEI, Hillsboro, OH, USA). The dispersions of Multi-walled CNTs (MWCNTs) were characterized using UV–vis spectrophotometer (Cary 4000, Varian, Palo Alto, CA, USA) operated with the ranges of 350–800 nm. Surface roughness and surface morphologies of CF were characterized by SEM (JCM-6000, JOEL, Tokyo, Japan) and AFM (XE-100, Park, Seoul, Korea). All AFM images of CF using the tapping mode obtained with the scan area of 4 mm × 4 mm. Surface roughness of the fibers were calculated from Equations (1) and (2) by the instrument software. At least 20 valid data points were applied for each specimen.

(1) $$R_{q}=\sqrt{\frac{1}{N^{2}}\sum_{i=1}^{N}\sum_{j=1}^{N}{\left(z_{ij}-z_{av}\right)}^{2}}$$

(2) $$R_{a}=\frac{1}{N}\sum_{i=1}^{N}\sum_{j=1}^{N}\left|z_{ij}-z_{cp}\right|$$

where $R_{q}$ is the root mean square (RMS) roughness, $R_{a}$ is the arithmetic mean roughness, *N* is the number of data points in the image, i and j are pixel locations on the AFM image, $z_{ij}$ is the height value at i and j locations, $z_{av}$ is the average height value within the given area, and $z_{cp}$ is the height value from the center plane.

The surface chemical properties of carbon fibers were evaluated by XPS with an Al Ka radiation (1486.6 eV) at a base pressure of 2 × 10^−9^ mbar. The XPS was energy referenced to the C1s peak at 284.6 eV. The XPS peak version 4.1 program was used for data analysis.

Dynamic contact angle tests were measured using a dynamic contact angle meter (DCAT21, Data-physics Instruments, Filderstadt, Germany). Deionized water ($\gamma^{d}=21.8\;{\mathrm{mNm}}^{-1}$, $\gamma^{p}=51.0\;{\mathrm{mNm}}^{-1}$, $\gamma =72.8\;{\mathrm{mNm}}^{-1}$) and glycol ($\gamma^{d}=29.3\;{\mathrm{mNm}}^{-1}$, $\gamma^{p}=19\;{\mathrm{mNm}}^{-1}$, $\gamma =48.3\;{\mathrm{mNm}}^{-1}$) were used as test liquids, the surface free energy, and its dispersive and polar components can be derived as the following equations:

$$\gamma_{l}\left(1+\cos\theta \right)=\frac{4\gamma_{l}^{d}\cdot \gamma_{s}^{d}}{\gamma_{l}^{d}+\gamma_{s}^{d}}+\frac{4\gamma_{l}^{p}\cdot \gamma_{s}^{p}}{\gamma_{l}^{p}+\gamma_{s}^{p}}$$

$$\gamma_{\mathrm{Total}}=\;\gamma_{s}^{d}+\;\gamma_{s}^{p}$$

where θ is the dynamic contact angle, $\gamma_{l}$, $\gamma_{l}^{d}$, and $\gamma_{l}^{p}$ is surface tension of the testing liquid, its dispersive, and its polar component. Each measurement was performed at least 10 times.

Interfacial property of CF/EP composite was evaluated by ILSS and water absorption. The ILSS of the CF/EP composites were measured by short beam shear tests according to American Society for Testing and Materials (ASTM) D2344. The tests were performed on a universal testing machine with a constant cross head rate of 2 mm/min and a span to thickness ratio of 5. The tests were carried out at 20 °C and 50% relative humidity. Specimen dimensions were 25 × 6 × 2 mm^3^, The ILSS was calculated as the following equation:

$$\tau \;=\;\frac{3P_{b}}{4b\cdot h}$$

where τ is the ILSS, $P_{b}$ is the maximum load, *b* and *h* are the width and the thickness of the specimen. All of the ILSS results were taken as the average value of more than five successful measurements.

Water absorption behaviors of CF/EP composite were researched to evaluate the influence of CNTs on interfacial adhesion of the composite. The test was carried out according to the ASTM D570 method. Percentage of water absorption can be calculated to the nearest 0.01% as follows:

(3) $$\mathrm{water}\;\mathrm{absorption}\;=(W_{t}-W_{0})/W_{0}\times 100\%$$

where $W_{t}$ and $W_{0}$ stand for the wet weight and the initial mass of the composite specimens, respectively.

## 3. Results and Discussion

### 3.1. Morphology and Dispersion of CNTs

**Figure 2:**
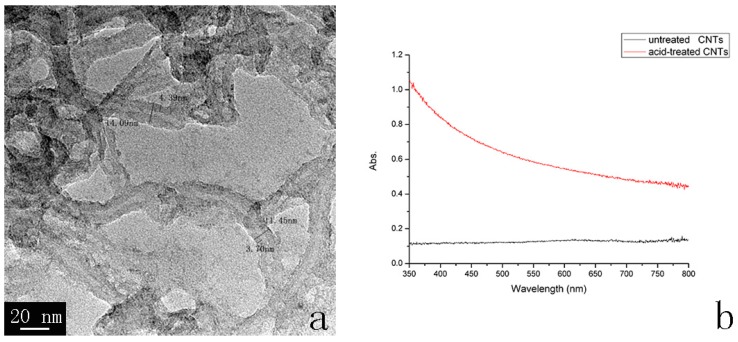
(**a**) TEM image of carbon nanotubes (CNTs); (**b**) UV–vis spectra of the untreated and base-treated CNTs.

To investigate the morphology of CNTs, TEM image of CNTs dispersion in water is shown in Figure 2a. As indicated in Figure 2a, the diameter of CNTs ranges from 10 to 20 nm and the wall thickness ranges from 3 to 5 nm, which reveals the CNTs have a multilayered structure and the graphitic sheets are almost parallel to the axial direction. UV–vis spectra of the untreated and base-treated CNTs in water are shown in Figure 2b. It can be seen from Figure 2b that base-treated CNTs show better dispersion than the untreated one, which ensures that the EPD process is effective.

### 3.2. Surface Roughness and Surface Morphologies of Carbon Fiber

**Figure 3:**
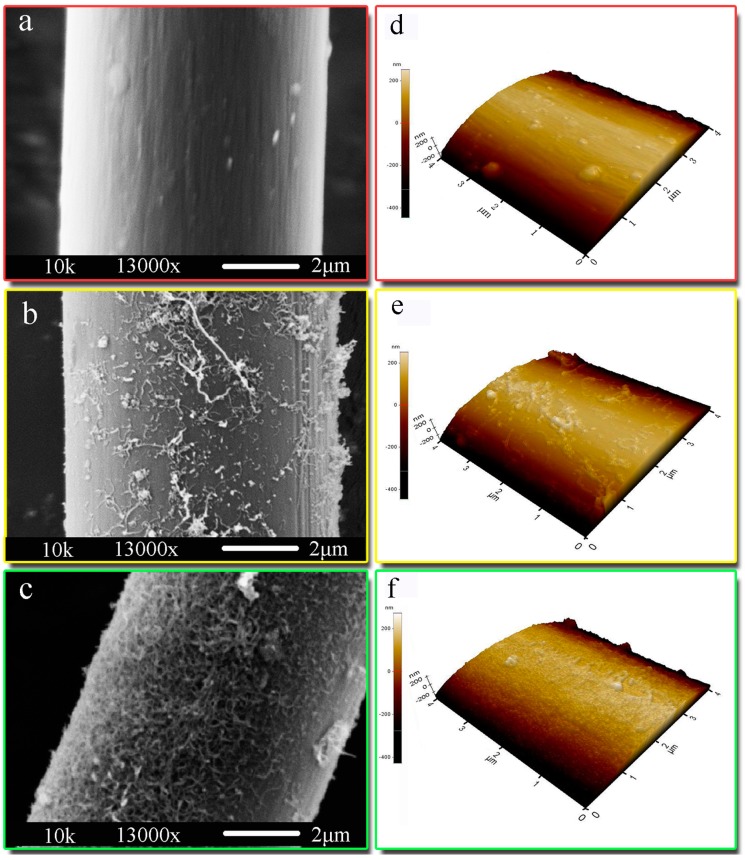
Scanning electron microscopy (SEM) and atomic force microscopy (AFM) images of carbon fiber (**a**,**d**) desized; (**b**,**e**) CNTs deposited without ultrasonic; (**c**,**f**) CNTs deposited with ultrasonic.

SEM images of CF at different stages were demonstrated in Figure 3. It was found that the desized CF had a smooth surface (Figure 3a). As shown in Figure 3c,d, CNTs were deposited on the surface of CF. It also shows that EPD process can prepare CNTs/CF hybrid fiber successfully. Meanwhile, homogeneous and thick CNT films due to ultrasonication can be seen from the SEM images of CNTs/CF hybrid fiber, the surface of CF with CNT deposits showing some slight concave pits and convex hills without ultrasonication (Figure 3b), whereas the surface of the CF showed much more convex hills and homogeneous coverage of the whole surface with ultrasonication (Figure 3c). It appears that ultrasonic during the EPD process can increase the quality and quantity of depositing CNTs.

In order to have better understand the surfaces of CF, AFM studies of the three samples with $R_{a}$ have been carried out. It can be found that desized CF (Figure 3d) shows a smooth surface with streaks, as shown in Figure 3e,f, the introduction of CNTs can increase the surface roughness of CF obviously. In addition, compared to CNT deposited CF without ultrasonication (Figure 3e), the surface roughness of CF with ultrasonically-assisted CNTs deposits showed an obvious increase (Figure 3f). The increase of surface roughness is beneficial for enhancing mechanical interlocking between the CF and EP [12]. Surface roughness of the carbon fiber, which can be characterized by arithmetic mean roughness $R_{a}$. After depositing CNTs with ultrasonication, a sharp increase in $R_{a}$ from 112.1 to 140.9 nm was detected. These results show that surface roughness and the morphologies of CF are enhanced, which can improve the wettability and interfacial adhesion between carbon fibers and matrix [16]. In addition, these results show that the introduction of ultrasonic of the CF during the EPD process increased the surface roughness of CF.

### 3.3. Surface Chemical Composition Analysis

**Figure 4:**
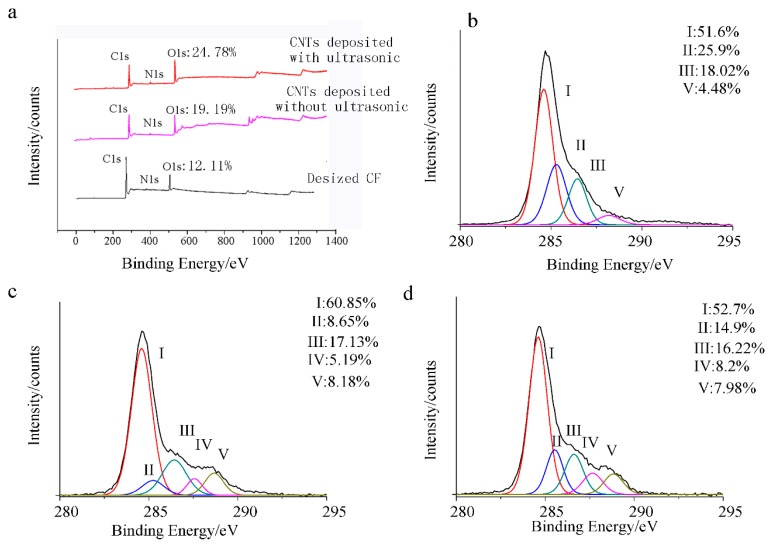
(**a**) The X-ray photoelectron spectroscopy (XPS) wide-scan spectra; (**b**) C1s spectra of desized carbon fiber (CF); (**c**) C1s spectra of desized CF without ultrasonic; (**d**) C1s spectra of desized CF with ultrasonic.

To further certify the chemical reaction of the whole depositing, XPS analysis was used to characterize surface chemical composition of CF and the results are compiled in Figure 4. High resolutions for C1s were performed to analyze the surface elemental state (Figure 4a–d), four peaks: graphitic carbon (peak I, 284.6 eV, peak II, 285.2 eV); carbon in phenolic, alcoholic, and ether groups (peak III, 286.1–286.3 eV); and carboxyl or ester groups (peak IV, 288.4–288.9 eV) were determined [19,20]. It was found that the carbon concentration declined sharply and the oxygenated groups concentrations of the CF surfaces experienced significant increases from 12.11% to 24.78% (Figure 4a), which suggests that the CNTs introduced some polar groups to CF surfaces. After depositing CNTs on the CF with ultrasonic, the concentrations of reactive oxygenated groups (IV) of CNTs/CF hybrid fiber are 8.2%, quite comparable with those of CNTs deposited without ultrasonic (5.19%), indicating that the introduction of ultrasonication of the CF during the EPD process increased the amount of CNT coatings.

### 3.4. Surface Wettability of Modified Carbon Fibers

The surface wettability of CF were characterized by DCAA. Table 1 shows the results of contact angles with water and glycol and surface free energy of desized CF and CNTs/CF hybrid fiber prepared by EPD-only and ultrasonically assisted EPD. The contact angles of water, the dispersive component of surface free energy, the polar component of surface free energy, and the total surface free energy on the desized CF were 76.07°, 9.54 mJ/m^2^, 23.18 mJ/m^2^, and 32.72 mJ/m^2^. In addition, after depositing CNTs on the carbon fiber with ultrasonication, the contact angles of water on CF reduced to 56.78°, the dispersive component of surface energy, the polar component of surface energy, and the total surface free energy increased to 10.58, 35.9 and 46.48 mJ/m^2^, this comes from two resources: the increasing surface area and polar groups due to the deposition of CNTs, which has a strong effect on the wettability of CF. Respectively, it can be also found that the free energy increased from 46.48 mJ/m^2^ for CNT deposited CF without ultrasonication to 50.55 mJ/m^2^ for CNT deposited CF with ultrasonication by 8.8%, the improvement in the surface wettability can be attributed to the reduction of the effect of hydrolysis caused by ultrasonication, which is beneficial to the deposition of CNTs on CF surface [15].

### 3.5. Interfacial Adhesion of the Composite

The interfacial adhesion between CF and EP resin was evaluated by ILSS and the water absorption. The water absorption of composites is not only related to the properties of EP and CF, but also with the porosity in the composite and the interfacial adhesion. Hygrothermal environments cause damage to the interface of the composite and reduce the mechanical properties of CF/EP composite [21,22,23]. Thus, the water absorption of composites indirectly reflect the interface bonding situation of composite.

**Figure 5:**
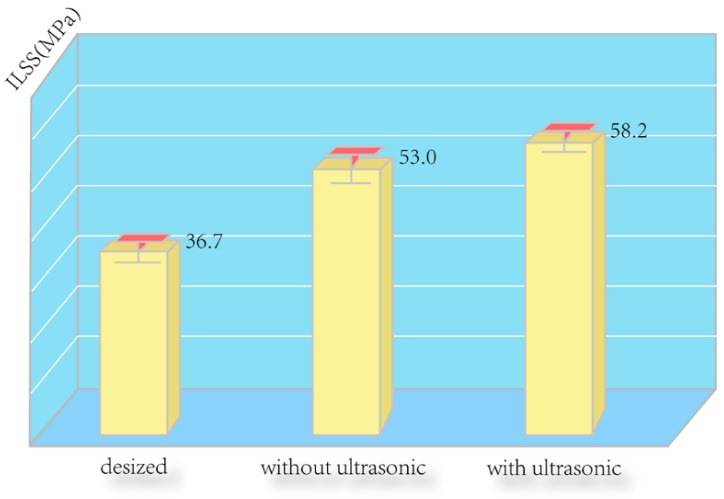
Interlaminar shear strength (ILSS) of the composites reinforced by the desized CF, CNTs deposited CF without ultrasonication, CNTs deposited CF with ultrasonication.

**Figure 6:**
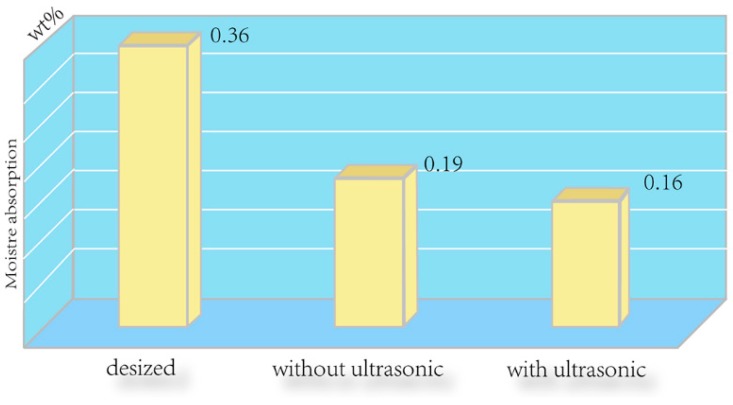
Water absorption of the composites reinforced by the desized CF, CNTs deposited CF without ultrasonication, CNTs deposited CF with ultrasonication.

The ILSS and water absorption results are given in Figure 5 and Figure 6, respectively. It is found that the ILSS and water absorption of the untreated composite were 36.7 MPa and 0.36%, respectively. However, after depositing CNTs on the fiber surface, the ILSS of the composite increased to 53.0 MPa with an increment of about 44.4%, while the water absorption of the composite declined to 0.19%. These results suggest that depositing CNTs on the CF surface improved the interfacial adhesion of the CF/EP composites by increasing surface roughness, which provided intimate molecular contact and stronger interfacial mechanical interlocking between the hybrid fiber and matrix. In addition, the oxygen-based functional groups of the CNTs are beneficial to enhance the interfacial adhesion by establishing hydrogen bonding with many sites in the epoxy resin during curing process [24]. After CNT deposition with ultrasonication, the ILSS further increased to 58.2 MPa with an increment of about 58.6%, and the water absorption of the composite degraded to 0.16%. These results come from the increase of the amount of CNTs caused by introduction of ultrasonication during the EPD process, which can serve as a new interface layer and further reduce the interlaminar stress concentration, enhance the strength and toughness of interfacial regions surrounding the CF, and finally lead to the improvement of the ILSS [25].

### 3.6. Fracture Mechanisms of the Composite

**Figure 7:**
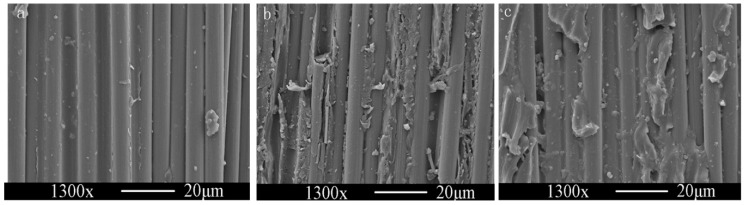
Fractured SEM images of CF/epoxy (EP) composite (**a**) desized CF; (**b**) CNT deposited CF without ultrasonication; (**c**) CNTs deposited CF with ultrasonication.

**Figure 8:**
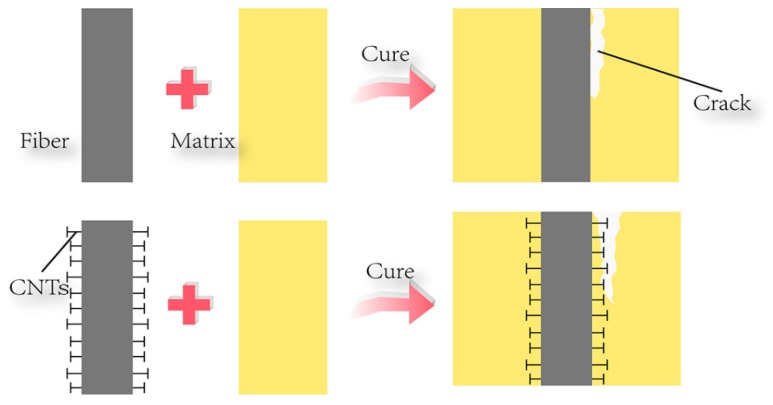
Schema for the differences between pristine and sonicated composites.

To help better understand the interface behavior and enhancement mechanism of CNTs/CF/EP composites, the SEM images of fractured surface of CF/EP composites is shown in Figure 7. For the composites without CNTs, the EP completely detached from the fiber surface because of a weak adhesion (Figure 7a), and the failure location was the interface between CF and EP resin. However, the fracture surface of the composites with CNT deposition is significantly different, with an amount of EP resin adhered to the CF surfaces (Figure 7b,c), the failure location between the interface and the EP. The improvement in the interfacial adhesion could be attributed to the new interface layer formed by depositing CNTs on the fiber surface. The strong mechanical interlocking changes the primary failure mode from interface failure to matrix fracture, which suggests that the interfacial adhesion of CF/EP composite was improved by depositing CNTs on the fiber surface. In addition, the ultrasonication is beneficial to enhance the interfacial adhesion by increasing the homogeneity of CNT film. Ultrasonication exorcises the tiny bubbles and inhibits the hydrolysis process through cavitation and emulsification impact [26]. It can be clearly seen that the disappearance of gradient interphase decrease the ILSS and flexural properties of hierarchical composites, which confirms the positive effect of gradient interphase on the interfacial properties from a different angle. These microcracks coalesce and propagate and the exact structure differences between the pristine, hierarchical, and sonicated composites are illustrated in Figure 8.

## 4. Conclusions

In the present work, CNTs were successfully coated onto CF surfaces via ultrasonically assisted electrophoretic deposition, which avoided complex chemical reactions and long processing duration. Meanwhile, the ultrasonication has significantly improved the surface performance of CF and the mechanical performance of CF/EP composites. SEM and AFM analysis showed that CNT film formed on carbon fibers and surface roughness of the CF was increased. XPS results confirmed ultrasonication can improve the amount of reactive oxygenated groups. DCAA results indicated that the deposition of CNTs significantly improved surface free energy of carbon fiber by increasing surface area and polar groups. Comparing the results with EPD-only, ultrasonically assisted EPD increased the thickness and uniformity of CNT coatings. This comes from ultrasonication reducing the effect of hydrolysis and exorcising the tiny bubbles. The ILSS was improved greatly with increasing amplitude of 58.6% due to interfacial mechanical interlocking between the hybrid fiber and EP. Fractured SEM images of CF reinforced composite suggested that the primary failure mode of CF/EP composites changed from interface failure to matrix fracture after the CF was treated. This newly proposed route for CF functionalization avoids complex chemical reactions and long processing duration and thereby provides great potential for industrial applications. It was also proved that CNT coating CF by EPD has negative effects on the mechanical properties of composites.

## Figures and Tables

**Table 1:** Contact angles and surface free energy.

Fiber type	$\theta_{\mathrm{water}}\;\left({}^{\circ}\right)$	$\theta_{\mathrm{glycol}}\;\left({}^{\circ}\right)$	$\gamma^{d}\;\left(\mathrm{mJ}/m^{2}\right)$	$\gamma^{p}\;\left(\mathrm{mJ}/m^{2}\right)$	$\gamma \;\left(\mathrm{mJ}/m^{2}\right)$
Desized CF	76.07	62.56	9.54	23.18	32.72
CNTs deposited without ultrasonication	56.78	47.43	10.58	35.90	46.48
CNTs deposited with ultrasonication	51.2	43.1	11.14	39.41	50.55
